# *In vitro* benchtop mock circulatory loop for heart failure with preserved ejection fraction emulation

**DOI:** 10.3389/fcvm.2022.910120

**Published:** 2022-07-22

**Authors:** Andrew Malone, Sean Gallagher, Jemil Saidi, Gina Rizq, Enda O’Dowd, Derek Vallence, Aamir Hameed

**Affiliations:** ^1^Tissue Engineering Research Group, Department of Anatomy and Regenerative Medicine, The Royal College of Surgeons in Ireland (RCSI), University of Medicine and Health Sciences, Dublin, Ireland; ^2^Medical Device Design, National College of Art and Design, Dublin, Ireland; ^3^School of Medicine, The Royal College of Surgeons in Ireland (RCSI), University of Medicine and Health Sciences, Dublin, Ireland; ^4^Trinity Centre for Biomedical Engineering, Trinity College Dublin, Dublin, Ireland

**Keywords:** Heart Failure with preserved Ejection Fraction (HFpEF), left atrium (LA), left ventricle (LV), mock circulation loop, electro-pneumatic regulator

## Abstract

In this work, a novel mock circulatory loop (MCL) is presented that is capable of simulating both healthy cardiac function and Heart Failure with preserved Ejection Fraction (HFpEF). This MCL differs from others presented in the literature as it features two independently actuated heart chambers, representing the left atrium and the left ventricle. This is an important improvement over other designs as it allows for potential HFpEF treatments to be examined, not just in relation to their effect on the left ventricle but also on the left atrium. The aim of this work was to show that novel MCL designs could be developed to allow for testing of new mechanical circulatory support devices for the treatment of HFpEF. Two loop configurations are presented, one featuring hard PVC cylindrical chambers and one that features soft silicone chambers which are anatomically analogous to the native heart. We show that both MCLs are capable of simulating the onset of HFpEF with a sustained increase in diastolic pressure of 62.03% and a sustained decrease in end diastolic volume (EDV) of 14.24%.

## Introduction

Heart Failure with Preserved Ejection Fraction (HFpEF) is one of two main phenotypes of Heart Failure (HF), the other being Heart Failure with Reduced Ejection Fraction (HFrEF). They are characterized by Left Ventricular ejection fraction (LVEF) ≥ 50% and LVEF ≤ 40%, respectively (1). HFpEF makes up approximately 50% of the HF population (2), a number that is expected to rise in the coming decades due to a combination of rising life expectancy, increased prevalence of metabolic disorders associated with this disease, and a lack of adequate therapies (2–6). This is despite the estimated economic burden associated with HFpEF predicted to $53.1 Billion in the United States by 2030 (7).

There is a clinical need for a robust *in vitro* testbed to develop technologies for the treatment of HFpEF. This is challenging as HFpEF is a multifactorial disease, typified by four phenotypes (8) which makes the simulation of the condition challenging. Previous attempts at developing a mock circulatory loop (MCL) have utilized single pneumatic chambers (9, 10), piston actuated *ex-vivo* hearts (11, 12), and external drive motors (13). The problems with *ex-vivo* heart models have been well documented; namely the rapid deterioration of electrophysiological and hemodynamic functions. One prominent *ex-vivo* model, the PhysioHeart™ (Life-Tec Group, The Netherlands) has been shown to be able to maintain homeostasis for just 171.4 ± 54 min (14). In addition, the use of *ex-vivo* and *in-vivo* models is becoming the subject of a growing ethical debate on animal rights, further complicating their use (15). This has led to the growing adoption of *in vitro* MCLs as alternatives to *in-vivo* and *ex-vivo* heart models (16).

Swier et al. developed one of the earliest *in vitro* cardiac models in 1989 (17). This model consisted of a 50-cc polyurethane right ventricle connected to a horse-shoe shaped blood reservoir which drove blood pneumatically. Ideally, an *in vitro* cardiac model should accurately mimic the physiological or pathological conditions of the human heart, including the tissue structure, extracellular matrix network, orientation, and circulation (18). We can further stipulate that a given model should capture the physiological markers and processes of disease where the model is designed for testing treatment options for said disease.

No *in vitro* testbed has been developed that can both mimic the cardiac cycle and features two independently controlled cardiac chambers to fully simulate the hemodynamics of the left atrium and left ventricle during diastole. A fully simulated left atrium is superior to a simple compliance chamber or preload reservoir as it can be used to monitor and actively control the left atrial pressure, an important physiological parameter in HFpEF. This is also important as the dynamics of HFpEF are complex and crucially, any treatment for HFpEF would necessarily be required to operate in tandem with both left heart chambers to function successfully (19). The requirement for a robust *in vitro* testbed necessitates a dynamic two-chambered MCL which can be altered to limit diastolic filling of the left ventricle while maintaining a functional cardiac cycle.

This work aims to present a new bench top MCL which can mimic both healthy cardiac function and HFpEF conditions through the limiting of diastolic relaxation.

## Materials and methods

**FIGURE 1:**
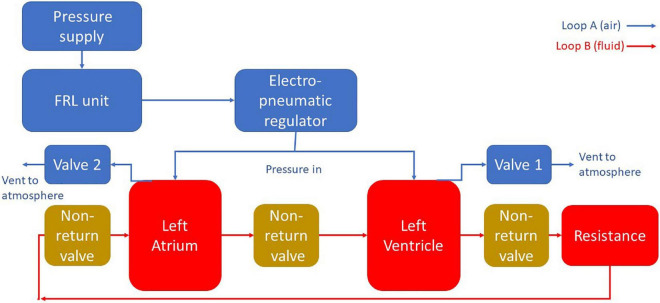
A schematic diagram of the MCL apparatus. Pressure is delivered to the chambers using pneumatic regulation. The flow in the blood loop is achieved by selectively pressurizing and depressurizing the heart chambers using solenoid valves. Backflow is prevented through the use of non-return valves.

For both designs the MCL was divided into two main sections. The “blood” loop, consisting of the left atrium, the left ventricle, and the vasculature (here meaning a simplified analog of the cardiovascular system outside of the left heart modeled using tubing and a tap to simulate peripheral resistance); and the air loop consisting of the pneumatic components to control the cardiac cycle. A schematic diagram of this apparatus is included in Figure 1.

The air loop for both MCLs consisted of a EMD400-41 pneumatic pressure supply (GCE Group Ltd., Sweden) connected *via* flexible PVC tubing (12 mm OD, Radionics Ltd., Ireland) to a filter, regulator, lubricator (FRL) unit consisting of an AMG350C-F04D water separator (SMC Pneumatics Corporation, Japan), an AF40-F04-A air filter (SMC Pneumatics Corporation, Japan), and an AR40-F04-1-B regulator (SMC Pneumatics Corporation, Japan). The FRL unit was then connected to an ITV1010-212BL5-X88 electro-pneumatic regulator (SMC Pneumatics Corporation, Japan) which delivered the final pressure into the system.

The electro-pneumatic regulator was set to deliver a pressure of 120 mm Hg through a T-junction to both cardiac chambers simultaneously. The chambers were also connected to two solenoid valves which were independently controlled using a transistor circuit and Arduino microprocessor (Arduino, Italy). By sending a signal *via* Arduino at a specific time interval, the two solenoids could be independently activated to vent their respective chamber, lowering the pressure, and cause blood to enter the chamber. When the solenoids deactivated the chamber repressurised and the blood was forced out, moving in a forward direction due to the presence of the PVC-U non-return valves.

### Mock circulatory loop mark I (Mk 1) production

The heart chambers of the Mk I were constructed of two vertical sections of acrylic pipe (38 mm OD × 32 m ID, Radionics Ltd., Ireland) while the vasculature was formed with a loop of the same piping while peripheral resistance was simulated using a PVC-U two-way ball valve (Georg Fischer Ltd., Switzerland). Three PVC-U ball non-return valves (Georg Fischer Ltd., Switzerland) were added to the blood loop to maintain forward blood flow. One was positioned before the left atrium to prevent backflow into the vasculature during atrial systole; the other two valves were positioned analogously to the mitral and aortic valves.

### Mock circulatory loop mark II (Mk II) production

Following initial testing with the MCL Mk I, work began on an updated MCL design which would incorporate anatomically realistic cardiac chambers, acquired from casting a porcine heart sourced in a local butcher. The MCL Mk I, while cheaper and easier to assemble, did not fully capture the anatomical geometry and structure of the heart, the updated Mk II design was developed to move closer to mimicking the situation *in vivo*. The internal space of the left ventricle was cast using room-temperature-vulcanizing (RTV) silicone. The silicone cast was used to model the geometry for a secondary cast made of Chavant modeling clay with a volume of 135 ml.

**FIGURE 2:**
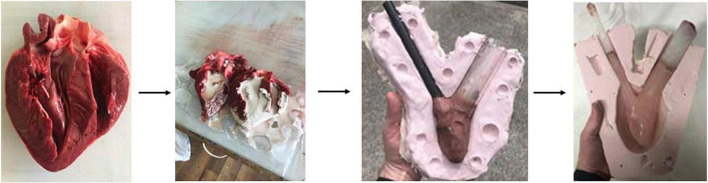
The internal volume of the left ventricle was cast using room-temperature vulcanizing silicone. Chavant clay was used with attached tubing to form the core for the reverse molding of the RTV silicone. The silicone outer mold was then used to rapidly produce multiple cores made of paraffin wax. Finally, the final chamber model was produced by pouring Platsil Gel 10 silicone into the interstitial space between the larger mold and the wax core.

The negative space of the aorta was modeled using the outer diameter of the acrylic tubing used in the MCL Mk I, and a secondary piece of tubing was added to model the negative space of a connecting tube between the ventricle and the atrium. This secondary tube was used because this design of the MCL necessitated that the atrium and the left ventricle be physically separated. Using the Chavant clay and tubing as a core, a two-part reverse mold was produced using RTV silicone which was used to produce multiple ventricle cores from paraffin wax, approximately 10 mm of water-based clay was added to the Chavant core in layers to represent the myocardium of the left ventricle. A large two-part silicone reverse mold was produced using this core to provide an outer surface for the finished chamber (Figure 2).

Each wax core was painted with a thin layer of Platsil Gel 10 silicone and left to cure. This thin layer of silicone ensured that the final piece was fully watertight as well as ensuring that the core was not in direct contact with the wall of the outer mold. The painted wax core was placed into the outer mold and Platsil Gel 10 silicone was poured into the interstitial space between the core and the mold. Once set, the silicone cast was removed and placed upside-down in an oven at 180°C. The wax core melted away after approximately 2 h, leaving only a hollow silicone ventricle model with two vessels representing the aorta and atrium attachment. The final dimensions of the silicone model were internal diameter = 5.9 ± 0.02 cm, internal length = 8.6 ± 0.02 cm, wall thickness = 1.0 ± 0.02 cm. To allow for two chambers to be operated, a second chamber was produced with a reduced internal volume to function as the left atrium. This was achieved in the same manner as the production of the ventricle but with a 15 mm layer of clay to create a smaller internal volume. The left atrium had dimensions of internal diameter = 4.8 ± 0.02 cm, internal length = 7.7 ± 0.02 cm, wall thickness = 1.6 ± 0.02 cm. The use of a second chamber provides a further advantage in that it can be modeled to represent the enlargement of the left atrium which is typically seen in HFpEF.

**FIGURE 3:**
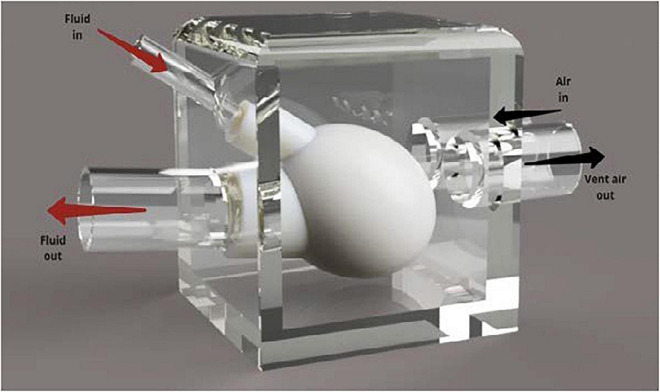
Annotated render of the silicone heart chamber inside a Tupperware container. The rigid plastic container was used to redirect the pressurized air to compress the heart chamber during systole.

To allow for compression of the silicone heart chamber, the model was placed in a modified plastic container. A render of this design can be seen in Figure 3. The plastic container was chosen because it was a readily available, airtight vessel with a removable lid that was large enough to house the silicone ventricle. It was important that the testing assembly included a lid so that the device could be adjusted during testing. Using forstner drill bits, two holes were drilled into the side of the container and PVC tubing that was positioned in the holes. The tubing was then secured in place using hot glue. The silicone blood vessels were then pushed over the PVC tubing and secured in place using cable ties. Two additional holes were drilled into the side of the box so that compressed air could be pumped into the box using the pneumatic system and vented through the solenoid valve, respectively. The container was pressurized to a pressure of 20 kPa (∼150 mm Hg) to overcome the stiffness of the silicone and deliver physiological pressure to the internal blood mimic.

Fluid enters the silicone left ventricle *via* the smaller diameter PVC inlet tube (15 mm). Compressed air is then injected into the box. This increased pressure causes the silicone ventricle to contract, forcing fluid out of the larger tube that represents the aorta. Backflow is prevented through the use of PVC-U non return valves as in the MCL Mk I design. During operation, air is vented out of the container, lowering the pressure, and allowing the silicone chamber to relax and refill with fluid.

### Measurement of cardiac parameters

The two cardiac parameters of interest modeled by the MCL were volume and pressure. Pressure was measured using two 24PCBFA6G gauge pressure sensors (Honeywell International Inc., United States) connected to two INA125 instrumentation amplifiers (Texas Instruments Inc., United States). The output from the pressure sensors was read using an Arduino and instantaneous pressure values could be recorded from both cardiac chambers simultaneously. The baud rate of the Arduino was set at 115,200 bits/s.

Graduation marks were placed on the side of the chambers to allow for qualitative estimates of maximum and minimum volumes to be made by eye but the measurement of volume in the cardiac chambers was not possible quantitatively. Instead, volume was determined indirectly using Doppler ultrasound. An acoustic blood mimicking fluid (BMF) was produced using Orgasol^©^ particles (2001UDNAT1, 5 μm diameter) based on work carried out by Ramnarine et al. (20). Three measurement sites were selected in the circulatory loop where blood velocities would be measured: immediately prior to the atrium (A), between the atrium and the ventricle (B), and immediately after the ventricle (C). Prior to measurement, the BMF was degassed, and the system allowed to run continuously for 1 h to ensure that no bubbles remained in the fluid.

A Logiq P6 ultrasound scanner (GE Healthcare, United States) with a broadband linear transducer (GE 11L) was used for determining the velocity of the BMF in pulsed wave spectral Doppler mode. A scanning well containing degassed water-glycerol (10% glycerol and 90% water) solution was used as an acoustic window between the transducer and the tubing. A beam-to-vessel angle of 60 was produced and the transducer face was positioned with a 20 mm layer of the water-glycerol solution separating it from the tubing. The B-mode image was adjusted to position the tubing in the center of the field of view, and the focal zone was set to the depth of the tubing in the middle of the image. The pulsed wave spectral Doppler mode was optimized by setting the Doppler gain to 65%, the Doppler range gate was set to 1 mm and positioned in the center of the tubing, the pulse repetition frequency was adjusted so that the Doppler spectrum encompassed 75% of the available spectrum window, and the wall filter was turned off. For each measurement site, five spectra were recorded of 4 s in duration each. These spectra were saved as JPEG files and exported to USB.

Custom code was developed in MATLAB (Mathworks, United States) to analyze the recorded Doppler spectra. First, the image was opened, and the user was asked to specify where the 0 and 100 cm/s graduations are in the image. This step had two purposes: the first was to calculate a pixel to velocity conversion factor and the second was to generate a scale bar in terms of pixels which the velocity spectrum will be compared against. A high pass filter was applied to the image to remove low level noise and isolate the pixel intensities of the spectrum. An edge detection algorithm was run on the image to trace the top of the velocity spectrum, which gave the maximum velocity with respect to time. The velocity information was then converted to volumetric flow rate by multiplying by the cross-sectional area of the tubing. Finally, the cumulative volume was determined using numerical trapezoidal integration on the volumetric flow rate. By comparing the cumulative volumes at the different measurement sites, cardiac chamber volumes could be determined as follows:


(1) $$V\,o\,l_{L\,A}=C\,u\,m\,V\,o\,l_{A}-C\,u\,m\,V\,o\,l_{B}$$

(2) $$V\,o\,l_{L\,V}=C\,u\,m\,V\,o\,l_{B}-C\,u\,m\,V\,o\,l_{C}$$

Where Vol_LA_ and Vol_LV_ are the volumes of the atrium and ventricle, respectively, and CumVol_A_, CumVol_B_, and CumVol_C_ are the cumulative volumes at measurement sites A, B, and C, respectively.

## Simulating heart failure with preserved ejection fraction

In both MCLs, it was possible to simulate heart failure with preserved ejection fraction (HFpEF) through the adjustment of the solenoid controls. HFpEF can be simulated through the following mechanism: As the diastolic phase of the cardiac cycle was initiated by venting pressure from the ventricle chamber itself (MCL Mk I) or the container housing the ventricle (MCL Mk II), reducing the amount of time that the solenoid valve was open for would result in an incomplete depressurization and insufficient ventricular filling.

This method of simulating HFpEF had the side effect of shortening the cardiac cycle as the length of diastole is decreased. In order to account for this when testing cardiac-gated medical devices, the response of the device must be adapted to match the change in heart rate. This was not seen as a problem for the testing of these devices as variable heart rate is to be expected *in vivo* and any device that is designed to operate at only certain points in the cardiac cycle should be capable of varying its duty cycle to match the current heart rate.

For both HFpEF and healthy cardiac function, the performance of each MCL was compared to each other using a paired *t*-test. The null hypothesis for this test was that the MCLs did not have any difference in performance at simulating both cardiac conditions and the alternative hypothesis was that the MCLs did perform differently. The significance level was set at *p* < 0.05.

## Results

**FIGURE 4:**
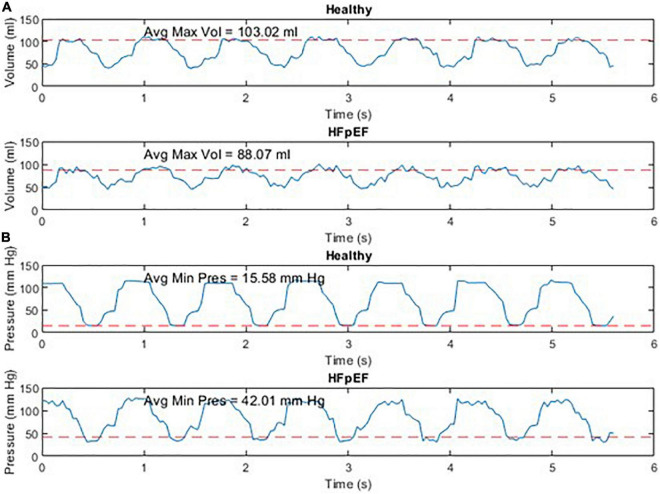
Instantaneous volume and instantaneous pressure for healthy cardiac function and for HFpEF for the Mk I MCL **(A)** and the Mk II MCL **(B)**.

**FIGURE 5:**
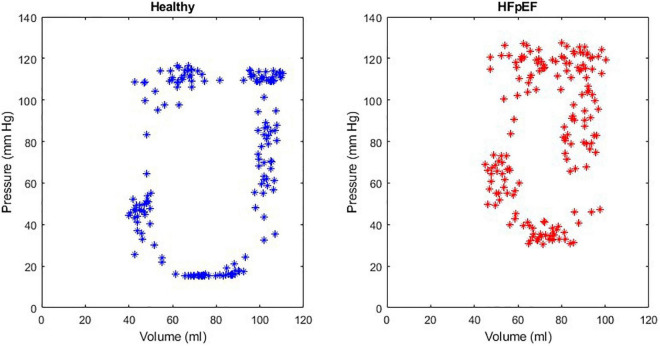
An example of a healthy PV loop and HFpEF PV loop generated from data recorded on the MCL Mk I.

**FIGURE 6:**
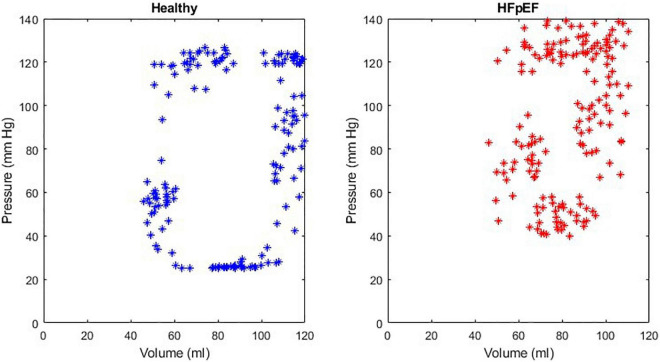
An example of a healthy PV loop and HFpEF PV loop generated from data recorded on the MCL Mk II.

An example of the instantaneous volume and instantaneous pressure curves for healthy heart function and for HFpEF for the Mk I and Mk II MCLs is included in Figure 4. An example of a pressure volume (PV) loop produced from the MCL Mk I for both health cardiac function and for HFpEF is included in Figure 5 and an example of a PV loop produced from the MCL Mk II for both health cardiac function and for HFpEF is included in Figure 6. The loops were produced by plotting the instantaneous pressure and volume against each other.

**FIGURE 7:**
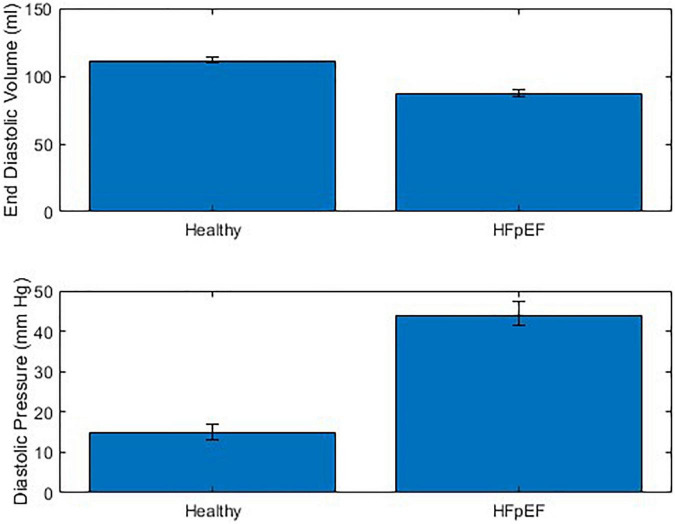
Combined performance of MCL Mk I and Mk II at simulating healthy cardiac function and HFpEF across 10 cardiac cycles (*N* = 30).

The performance of the MCL Mk I and Mk II was found to not be significantly different using a paired *t*-test (*p* = 0.60). The overall performance of both MCLs is presented in Figure 7 which shows the difference in End Diastolic Volume (EDV) and diastolic pressure averaged over ten cardiac cycles between healthy function and HFpEF in the MCLs. It can be seen in Figure 7 that when the reduction in diastolic duration is implemented, the average maximum volume decreases by 14.24% and the average minimum pressure increases by 62.03%. These values can be compared to values for end diastolic pressure and volume in the literature: The expected EDV decrease with HFpEF is from 121 (101–132) to 101 (86–105) as measured using echocardiography and the expected end diastolic pressure increase with HFpEF is from 8.0 (7.0–10.9) to 14.1 (10.6–18.0) (21). This is a volume decrease of 16.5% and a pressure increase of 43.26%.

While the MCL Mk II showed comparable results to the MCL Mk I but required additional pressure applied to the heart chambers to result in the same internal pressure being applied to the blood. This was hypothesized to be due to the silicones inherent elasticity resisting the compression from the external pressure. For physiological pressure ranges, the required additional pressure was found to be a linear relationship and for all pressure values, a flat additional 10 mm Hg was sufficient to overcome the elasticity of the silicone.

An additional issue was noted with the MCL Mk II caused by the geometry of the silicone; because the chamber was modeled on cardiac anatomy but did not contain any cardiomyocytes, the thickness of the silicone was not proportional to the local cardiac contraction and was instead inversely proportional. This is due to the fact that *in vivo*, the thickest part of the myocardium is usually the strongest, whereas when using inert silicone, the thickness adds to the chambers overall elasticity and reduces overall compression. This meant that higher external pressures were required to compress the thickest parts of the cardiac chamber.

## Discussion

A new MCL was presented for the purpose of early testing and validation of novel technologies for the treatment of HFpEF that interact only with the left side of the heart, such as circulatory support devices providing cannulation between the left atrium and left ventricle. Examples of devices which could be tested in this way are the mechanical circulatory support devices outlined by Rosalia et al. (1). Two models of the MCL were developed and assessed for their abilities to simulate HFpEF. The first MCL used vertical PVC columns as heart chambers and demonstrated the ability to adequately model both healthy cardiac function and HFpEF, while the second MCL used silicone heart chambers encased in plastic container to direct the applied pneumatic pressure. The MCLs were shown to not be significantly different in terms of performance and demonstrated a sustained increase in diastolic pressure of 62.03% and a sustained decrease in EDV of 14.24%. These results can be compared to literature values for end diastolic pressure and volume of 43.26 and 16.5%, respectively.

Miyagi et al. developed a similar MCL to that presented in this work for testing of the Left Atrial Assist Device (LAAD) for the treatment of HFpEF (9). In this study, Miyagi et al. used a mock ventricle (AB5000, ABIOMED Inc., Danvers, MA) in conjunction with a static blood reservoir representing the left atrium, and adjustable afterload and compliance simulators. Despite describing the AB5000 as a mock ventricle, the system is intended for use as a ventricular assist device, designed to provide temporary support to one or both sides of a patient’s heart. While we could expect that output of this device to be broadly equivalent to that of an *in vivo* heart, it cannot be considered analogous to the human heart. Furthermore, the lack of an independently operating left atrium limits the testbed’s ability to discern any treatments effect on the left atrium during its own cardiac cycle. Fukamachi et al. developed a similar MCL to Miyagi et al. using the same AB 5000 mock ventricle and static blood reservoir to represent the left ventricle and left atrium, respectively [Fukamachi et al., (10)]. Again, the issue with this design is the lack of any independent control over the left atrial pressure and the inability to examine any HFpEF treatments effect on the atrial cardiac parameters throughout diastole.

Leopaldi et al. developed a MCL using an *ex vivo* porcine heart model (11). The advantages of using an *ex vivo* model are clear, it allows for the most accurate representation of anatomical structures possible while still not having the same complexities associated with a full animal model. The primary disadvantage of using an *ex vivo* model is the limited lifespan of the model and requirement of replacing the model as the tissue degrade. This can lead to an inconsistent model geometry as models are replaced or inaccurate results due to altered mechanical properties in situations when *ex vivo* tissue is preserved to increase its lifespan. Furthermore, the model developed by Leopaldi et al. has a similar issue as discussed previously with Miyagi et al. and Fukamachi et al. namely the lack of an independently operated left atrium. In this study, the *ex vivo* heart is connected hydraulically to a piston pump *via* an apical connector and a preload reservoir representing the left atrium *via* an atrial connector. The atrial preload in this model is a constant, determined by the height of fluid in the reservoir. This makes it impossible to examine the performance of any HFpEF treatment in conjunction with atrial systole, which forms a key part of the left atrial hypertension seen alongside HFpEF (1).

Leopaldi et al. updated the MCL developed in a later work (12). This model again features an *ex vivo* porcine heart, which has been encased in a fluid filled chamber with a hydraulically connected piston pump. This model used a vacuum seal to ensure that the left ventricle could be isolated for independent actuation by the piston but again used a preload reservoir in place of the left atrium.

Liu et al. developed a MCL using a soft silicone model of the left ventricle based on CT images of a patient heart and thoracic aorta (13). The silicone model was placed inside an acrylic chamber and driven using an electric motor, although details on how this is achieved (be it direct actuation, pneumatic compression, or another method) are not given in the paper. This model is the similar to the MCL Mk II developed in this work and features a high degree of anatomical realism, although the study authors do note that they could not match the geometry of the native heart while it is pulsing. The MCL Liu et al. developed still lacks a left atrium and simply uses a preload reservoir as others have done. This again, limits the MCLs ability to examine any HFpEF treatment option in relation to the left atrium during diastole.

The literature on the development of MCLs shows a variety of approaches to mimic the anatomical structure of the heart for testing of HFpEF treatment options, however, most work in this space is subject to the same issue, a lack of an independently controlled left atrium and left ventricle. This limits any MCLs ability to assess the effectiveness of proposed treatment options with regard to the left atrium and its pressure-volume relationship through the cardiac cycle but specifically during ventricular diastole and atrial systole.

A major limitation of this work was a lack of raw hemodynamic data to directly compare the performance of the MCLs to the conditions *in-vivo*. Although the authors could not acquire raw pressure and volume data to provide a direct comparison across the cardiac cycle, it was possible to compare values at the key time point of end-diastole. At this time point, the percentage decrease in volume and percentage increase in pressure were broadly in agreement, however, the magnitude of the pressure change was larger for the MCLs. This discrepancy is likely due to the peripheral resistance being too high, resulting in a higher pressure needed to achieve the required blood volume. A solution to this would be the use of larger diameter tubing as well as a reduction in the LV afterload applied in the experiment.

Another limitation was the requirement that HFpEF be implemented by shortening the diastolic filling duration during the cardiac cycle. This resulted in an elevated heart rate and prevented a direct comparison of the HFpEF and healthy systems with an equal heart rate without also varying the durations of other periods in the cardiac cycle for the healthy system. This problem could be solved in several ways: (1) by lengthening the other periods of the cardiac cycle proportionally, losing a temporally analogous heart rate but maintaining the relative intervals, (2) using pulse wave modulation (PWM) functionality and a compatible solenoid, the valve to the left ventricle could be only partially opened, reducing the pressure reduction achieved in the same time interval, (3) in the case of the Mk II rig, the compliance of the chamber could be reduced while keeping the cardiac cycle timings unchanged.

A further limitation is the inability to adequately model the heterogenous population of HFpEF patients. Future work in this space would need to more accurately model the peripheral impedance of patients and perhaps more extensively model the cardiovascular system.

Another limitation of this work was the requirement for a specially formulated BMF to allow ultrasonic measurement of flow and determine chamber volume from the flow differences. A simpler, albeit more expensive method would be the use of flow probes which use time of flight measurements to determine velocity (and can be calibrated for tube size to output flow rate) and do not require specific BMF.

## Conclusion

The MCLs presented in this work are both are capable of simulating health heart function and can provide a method of mimicking HFpEF. Future iterations of this design have the potential to allow for full simulation of the spectrum of HFpEF severity. They both feature two chambers which can be independently controlled using a single pneumatic pressure source. They represent a key step forward in the development of a robust *in vitro* testbed for HFpEF treatments as well having potential for the simulation of other cardiac conditions which affect both the atria and the ventricles.

## Data availability statement

The original contributions presented in this study are included in the article/supplementary material, further inquiries can be directed to the corresponding author.

## Author contributions

AH, EO’D, and DV supervised and critically reviewed the manuscript. AM designed the *in vitro* rig and took a lead in writing the manuscript. SG developed the silicone heart model. JS and AM performed the experiments. JS and GR did the literature search and contributed toward writing the manuscript. All authors contributed to the article and approved the submitted version.

## Conflict of interest

The authors declare that the research was conducted in the absence of any commercial or financial relationships that could be construed as a potential conflict of interest.

## Publisher’s note

All claims expressed in this article are solely those of the authors and do not necessarily represent those of their affiliated organizations, or those of the publisher, the editors and the reviewers. Any product that may be evaluated in this article, or claim that may be made by its manufacturer, is not guaranteed or endorsed by the publisher.
